# Visual categorisation of the arch index: a simplified measure of foot posture in older people

**DOI:** 10.1186/1757-1146-5-10

**Published:** 2012-07-03

**Authors:** Hylton B Menz, Mohammad R Fotoohabadi, Elin Wee, Martin J Spink

**Affiliations:** 1 Musculoskeletal Research Centre, Faculty of Health Sciences, La Trobe University, Bundoora, Victoria, 3086, Australia

## Abstract

****Background**:**

Foot posture is considered to be an important component of musculoskeletal assessment in clinical practice and research. However, many measurement approaches are not suitable for routine use as they are time-consuming or require specialised equipment and/or clinical expertise. The objective of this study was therefore to develop and evaluate a simple visual tool for foot posture assessment based on the Arch Index (AI) that could be used in clinical and research settings.

****Methods**:**

Fully weightbearing footprints from 602 people aged 62 to 96 years were obtained using a carbon paper imprint material, and cut-off AI scores dividing participants into three categories (high, normal and low) were determined using the central limit theorem (i.e. normal = +/− 1 standard deviation from the mean). A visual tool was then created using representative examples for the boundaries of each category. Two examiners were then asked to use the tool to independently grade the footprints of 60 participants (20 for each of the three categories, randomly presented), and then repeat the process two weeks later. Inter- and intra-tester reliability was determined using Spearman’s rho, percentage agreement and weighted kappa statistics. The validity of the examiner’s assessments was evaluated by comparing their categorisations to the actual AI score using Spearman’s rho and analysis of variance (ANOVA), and to the actual AI category using percentage agreement, Spearman’s rho and weighted kappa.

****Results**:**

Inter- and intra-tester reliability of the examiners was almost perfect (percentage agreement = 93 to 97%; Spearman’s rho = 0.91 to 0.95, and weighted kappas = 0.85 to 0.93). Examiner’s scores were strongly correlated with actual AI values (Spearman’s rho = 0.91 to 0.94 and significant differences between all categories with ANOVA; *p* < 0.001) and AI categories (percentage agreement = 95 to 98%; Spearman’s rho = 0.89 to 0.94, and weighted kappas = 0.87 to 0.94). There was a slight tendency for examiners to categorise participants as having higher arches than their AI scores indicated.

****Conclusions**:**

Foot posture can be quickly and reliably categorised as high, normal or low in older people using a simplified visual categorisation tool based on the AI.

## **Background**

Measurement of foot posture is widely considered to be an important component of musculoskeletal examination in clinical practice and research, as variations in foot posture have been found to influence lower limb gait kinematics [1,2], muscle activity [3], balance and functional ability [4,5], and predisposition to overuse injury [6-8]. Unfortunately, there remains considerable disagreement regarding foot posture categorisation as several techniques have been reported in the literature, including visual observation [6,9,10], footprint parameters [11,12], measurement of frontal plane heel position [13,14], assessment of the position of the navicular tuberosity [15,16] and a range of angular measurements obtained from foot radiographs [17,18]. Each of these techniques has advantages and disadvantages in relation to equipment requirements, the degree of clinical expertise necessary to obtain accurate measurements, reliability and validity considerations, relationship to dynamic foot function and the availability of normative data for comparison purposes [19].

In 1987, Cavanagh and Rogers [11] developed the Arch Index (AI), which represents the ratio of the area of the middle third of a footprint relative to the total area excluding the toes, with a higher ratio indicating a flatter foot. The AI has since been found to have excellent reliability [20,21], is highly correlated with navicular height [20,22] and angular measures [20,23,24] determined from radiographs, is sensitive to age-related differences in foot posture [25], and is correlated with pressures under the midfoot [26-28] and rearfoot motion [29,30] when walking. However, the main drawback of the AI as a measure of foot posture is that it requires the use of a graphics tablet or optical scanner and imaging software in order to accurately calculate the footprint area, which is time-consuming and therefore limits its application in many clinical and research settings.

A simplified version of the AI not requiring computerised measurement or clinical expertise would be of practical value for clinicians and researchers seeking a reliable and valid measure of foot posture. Therefore, the aim of this study was to develop a simple visual categorisation tool based on the AI which allows foot posture to be documented into three categories (high, normal and low), and to evaluate the tool’s inter- and intra-tester reliability and validity in a sample of older people.

## **Methods**

### **Development of the visual AI tool**

In order to develop reference values for the determination of cut-off scores defining high, normal and low AI categories, previously collected AI scores were pooled from 602 participants aged 62 to 96 years (mean 75.7, SD 6.7). These participants were drawn from three different sources: a retirement village (n = 176), a database of people attending a university health sciences clinic (n = 121) and participants involved in a randomised controlled trial of a podiatry intervention to prevent falls (n = 305). Participant characteristics for each of these studies are provided in detail elsewhere [31-33], however for all three studies, the key exclusion criteria were an inability to walk household distances without the use of a walking aid, or cognitive impairment, defined as a score of less than 7 on the Short Portable Mental Status Questionnaire [34].

**Figure 1:**
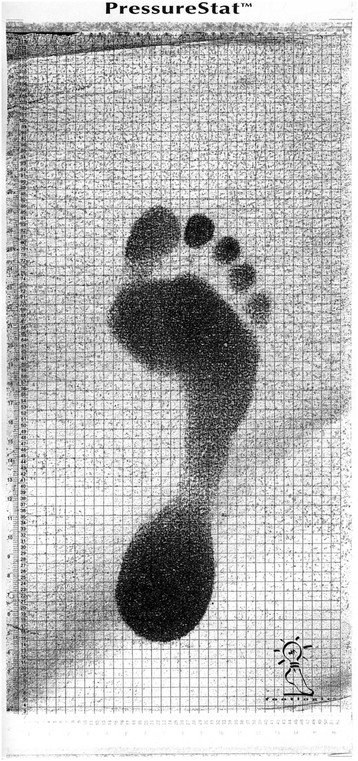
Footprint obtained using carbon paper imprint material.

**Figure 2:**
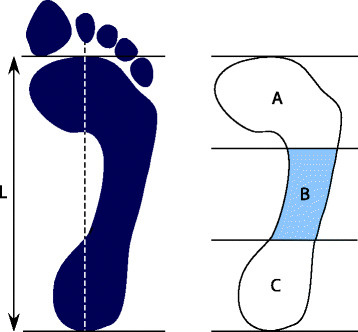
**Calculation of the AI.** The length of the footprint excluding the toes (L) is divided into equal thirds. The AI is then calculated as the area of the middle third of the footprint divided by the entire footprint area (AI = B/A + B + C).

In each of these studies, AI was determined by obtaining a fully weightbearing static footprint using carbon-paper imprint material (PressureStat™, FootLogic Inc., South Salem, NY, US) with the participant standing in a relaxed position (Figure 1). A foot axis was then drawn from the centre of the heel to the tip of the second toe, and the footprint divided into equal thirds (excluding the toes) by constructing lines tangential to the foot axis. Using a computer graphics tablet (Wacom Technology Corp., Vancouver, Canada) and graphics software (Canvas 8.0, ACD Systems, Miami, FL, USA), the AI was calculated as the ratio of area of the middle third of the footprint to the entire footprint area. The lower the arch, the higher the AI [11]. See Figure 2.

**Figure 3:**
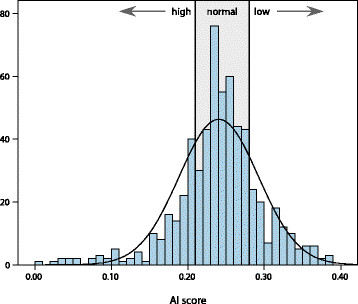
**Histogram of AI scores obtained from 602 people aged 62 to 96 years.** Cut-off scores defining high and low-ached feet based on ± 1 SD are indicated.

**Figure 4:**
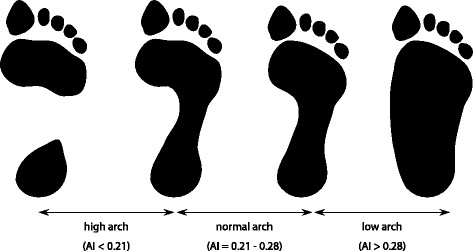
The visual AI categorisation tool.

AI scores ranged from 0 to 0.39 (mean 0.24, median 0.24, standard deviation [SD] 0.06) and were normally distributed (Figure 3), Three categories were created: normal (± 1 SD from the mean), high (<1 SD) and low (>1 SD). The AI scores that defined each category were as follows: normal (0.21 to 0.28), high (<0.21) and low (>0.28). A visual tool was then created using representative examples for the cut-off scores for each category. To ensure that examiners using the technique focused on the contours of the footprint, the selected footprints were resized to standard dimensions and provided with identical toe prints (see Figure 4).

### **Reliability evaluation**

AI data for the reliability component of this study were drawn from the 305 randomised controlled trial participants [35]. All participant’s AI scores were categorised as described above, and 60 footprints (20 footprints from each of the three categories: normal, high and low) were randomly selected. Two examiners – a physiotherapist with 22 years clinical experience (MRF) and a physiotherapist with 10 years of clinical experience (EW) – independently rated the footprints and were asked to categorise them as normal, high or low using the visual tool shown in Figure 4. The examiners then repeated their assessments two weeks later without reference to their baseline scores. The Human Ethics Committee of La Trobe University approved the study (ID: 07–118) and participants provided written informed consent.

### **Statistical analysis**

All analyses were performed using SPSS Statistics version 17.0 (SPSS Inc, Chicago, IL) and STATA version 8.2 (STATA Corp, College Station, TX). Statistical analysis was undertaken in two stages. Firstly, inter- and intra-examiner reliability was determined using percentage agreement, Spearman’s rho (ρ) and the weighted kappa statistic (κ_w_), which is considered to be the most appropriate statistic to assess the level of agreement when the measurement scale is ordinal. In contrast to the “standard” κ described by Cohen [36], κ_w_ also takes into account that the relative importance of disagreement between categories may not be the same for adjacent categories as it is for distant categories. For example, if one examiner documented the AI as normal while the other documented it as low, the κ_w_ approach would consider this to be less of an error compared to one examiner documenting it as high and the other documenting it as low. A quadratic assignment of weights described by Fleiss [37] was applied, and the following benchmarks for interpretation of κ_w_ scores were used: ≤0 = poor, 0.01 to 0.20 = slight, 0.21 to 0.40 = fair, 0.41 to 0.60 = moderate, 0.61 to 0.80 = substantial, and 0.81 to 1.00 = almost perfect [38].

Secondly, to determine the validity of the examiners’ assessments, their categorical AI scores were compared to the “gold standard” continuous AI scores obtained with the computerised graphics tablet using Spearman’s ρ and a one-way analysis of variance and Bonferroni post-hoc tests, and the categorical AI scores obtained with the computerised graphics tablet using percentage agreement, Spearman’s ρ and the κ_w_ statistic.

## **Results**

### **Inter-tester reliability**

The level of agreement between examiners was almost perfect for both session 1 (percentage agreement = 95%; ρ = 0.93, *p* < 0.01; κ_w_ = 0.89, 95% confidence interval [CI] 0.80 to 0.93) and session 2 (percentage agreement = 93%; ρ = 0.91, *p* < 0.01; κ_w_ = 0.89, 95% CI 0.80 to 0.93).

### **Intra-tester reliability**

The level of agreement between sessions was almost perfect for both examiner 1 (percentage agreement = 95%; ρ = 0.94, *p* < 0.01; κ_w_ = 0.89, 95% CI 0.83 to 0.95) and examiner 2 (percentage agreement = 97%; ρ = 0.95, *p* < 0.01; κ_w_ = 0.93, 95% CI 0.92 to 0.96).

### **Validity of examiners’ assessments compared to computer graphics tablet AI scores**

**Figure 5:**
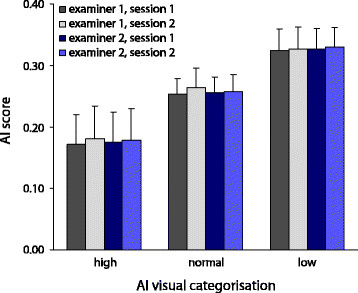
Mean (± SD) AI scores obtained by the computer graphics tablet according to each examiners’ categorisations in session 1 and 2.

Mean (SD) AI scores calculated using the computer graphics tablet across each of the AI categories documented by each examiner in each session are shown in Figure 5. There were significant differences in mean AI scores obtained using the graphics tablet across the AI categories documented by examiner 1 in session 1 (F_2_ = 85.6, *p* < 0.001), examiner 1 in session 2 (F_2_ = 62.7, *p* < 0.001), examiner 2 in session 1 (F_2_ = 80.9, *p* < 0.001) and examiner 2 in session 2 (F_2_ = 74.3, *p* < 0.001). All Bonferroni *post-hoc* tests across AI categories were significant at the *p* < 0.001 level.

The level of agreement between AI categories derived from the computer graphics tablet scores and examiners’ categories was very high for examiner 1, session 1 (percentage agreement = 98%; *ρ* = 0.94; κ_w_ = 0.94, 95% CI 0.88 to 0.94), examiner 1, session 2 (percentage agreement = 95%; *ρ* = 0.89; κ_w_ = 0.87, 95% CI 0.85 to 0.90), examiner 2, session 1 (percentage agreement = 97%; *ρ* = 0.92; κ_w_ = 0.92, 95% CI 0.85 to 0.95), examiner 2, session 2 (percentage agreement = 96%; *ρ* = 0.90; κ_w_ = 0.89, 95% CI 0.88 to 0.97).

The frequency of mismatches between the AI categories derived from the computer graphics tablet scores and examiners’ categories in each session are shown in Table 1.

**Table 1:** Frequency of misclassifications between the AI categories derived from the computer graphics tablet scores and examiners’ categories in each session

	n (%) correct observations	n (%) misclassifications, real AI higher than examiner’s score	n (%) misclassifications, real AI lower than examiner’s score
Examiner 1			
Session 1	55 (92)	4 (7)	1 (2)
Session 2	53 (88)	6 (10)	1 (2)
Examiner 2			
Session 1	49 (82)	10 (17)	1 (2)
Session 2	51 (85)	8 (13)	1 (2)

## **Discussion**

The objectives of this study were to develop a visual assessment tool based on the AI to enable foot posture to be easily categorised in older people, and to evaluate its reliability and convergent validity. The tool performed very well, with AI categories demonstrating almost perfect inter- and intra-examiner reliability and exhibiting strong associations with both continuous and categorical AI scores obtained with a computer graphics tablet (the “gold standard” for this measure). These findings suggest that it may not be necessary to perform the time-consuming task of measuring footprint surface areas in order to classify foot posture using the AI in clinical and research settings.

Before discussing these findings in detail, it is important to note that the cut-off scores we used to define each foot type category differ (albeit only slightly) to those originally proposed by Cavanagh and Rodgers [11], due to differences in sample characteristics and the statistical approach used. In the Cavanagh and Rodgers [11] study, AI scores were obtained from 107 young adults (mean age 30 years) without foot symptoms, resulting in a mean AI of 0.23 (SD = 0.04, range 0 to 0.36). Rather than using the traditional criteria of ± SD to define “normal”, Cavanagh and Rodgers [11] instead used quartiles, thereby creating a normal subgroup of participants representing 50% of the sample. Based on this approach, a low AI (indicative of a flatter foot) was defined as >0.26 and a high AI (indicative of a highly arched foot) was defined as <0.21. Our sample was larger (n = 602), older (mean age 76 years) and included participants with and without foot symptoms, which may explain our larger range of AI scores (0 to 0.39). In addition, we defined normal based on the conventional ± 1 SD criterion, thereby creating a normal subgroup of approximately 68% of the sample. Despite these differences, the mean AI in our study was similar (0.24), as were the cut-off scores (low AI >0.28 and high AI < 0.21).

Although the examiners’ AI categories correlated very strongly with the AI scores obtained with the computer graphics tablet, some degree of misclassification did occur (see Table 1). Specifically, there was a tendency for examiners to categorise participants as having higher arches than their AI scores indicated, with between 80 and 90% of misclassifications being caused by the assessor documenting the AI category lower than the AI category determined from the graphics tablet. This is not surprising, as the visual tool depicts the footprint in black and white, whereas the carbon paper imprint material is pressure-sensitive and therefore records gradations of contact between the foot and the supporting surface (see Figure 1). The degree of contact is particularly indistinct in the medial arch region, so when comparing the imprint to the visual tool, the examiners may have assumed that slight contact was no contact, thereby offsetting the AI classification towards a higher arch. Nevertheless, we believe that the degree of misclassification is within acceptable limits, given the high overall percentage agreement.

Based on our findings, it would appear that the AI visual assessment tool is worthy of consideration when selecting a foot posture measurement in clinical practice or research settings, as it overcomes the previous disadvantage of requiring a graphics tablet and software. The AI also offers some key advantages over other clinical measurements, as it is highly reliable [20,21], is correlated with navicular height [20,22] and angular medial arch measures [20,23,24] determined from radiographs, is correlated with pressures under the midfoot [26-28] and rearfoot motion [29,30] during gait, and is able to discriminate between foot types based on age [25] and presence of musculoskeletal conditions such as plantar fasciitis [39], midfoot osteoarthritis [32] and medial compartment knee osteoarthritis [40].

However, the validity of the AI as a measure of foot posture has been questioned by Wearing et al. [41], who suggested it may be a measure of “fat” feet rather than “flat” feet. This criticism is based on their finding of a significant association between AI and fat mass percentage in 24 overweight and obese individuals. Unfortunately, no measures of foot posture or arch height were collected in their study, so the relative associations between these variables could not be evaluated. Nevertheless, this finding, along with a more recent study reporting an association between AI and body mass index in older people [42], suggest that adiposity may influence the shape of the middle third of the footprint, particularly in overweight or obese individuals. Therefore, comparisons of AI scores between groups may need to consider body composition as a potential confounding factor, as recently demonstrated in study comparing AI in people with and without knee osteoarthritis [40].

There are three additional limitations to our study that require consideration. First, the tool was developed using a large dataset of older people. Older people have been shown to have flatter feet than young people [25], suggesting that the cut-off scores may not be valid for a younger group. However, the cut-off score for categorising a highly arched foot (0.21) was identical to the original description by Cavanagh and Rodgers [11], and the flat-arched foot cut-off score was only slightly higher (0.28 compared to 0.26). Nevertheless, this difference needs to be considered as some degree of misclassification (in the direction of a higher-arched foot) may occur if the tool is applied to a younger sample. Second, the two examiners we used had recent experience in clinical assessment of the foot as they had been responsible for data collection of the 305 older people in the clinical trial [33]. Although they had not used the visual AI tool before, their level of experience in foot assessment may have been at least partly responsible for the high level of reliability we found. Therefore, further research is required to examine reliability in less experienced examiners. Finally, the AI tool only provides a simple three-group categorisation of foot posture, so other foot assessments (such as the Foot Posture Index [10,43] and foot mobility [44,45]) may be more appropriate in situations where a greater degree of discrimination is required.

## **Conclusions**

Foot posture can be quickly and reliably categorised as high, normal or low in older people using a simplified visual categorisation tool based on the AI. The tool may therefore be useful for musculoskeletal screening in clinical practice or research settings where more detailed assessments of foot posture are not feasible.

## **Competing interests**

HBM is Editor-in-Chief of *Journal of Foot and Ankle Research*. It is journal policy that editors are removed from the peer review and editorial decision making processes for papers they have co-authored. The other authors declare that they have no competing interests.

## **Authors’ contributions**

HBM conceived the idea for the study, conducted the statistical analysis and drafted the manuscript. MRF, EW and MJS collected and compiled the data and assisted with interpretation of the data and drafting of the manuscript. All authors read and approved the final manuscript.
